# Anterior Segment Surgery Performed During the COVID-19 Pandemic

**DOI:** 10.14744/bej.2021.20092

**Published:** 2021-12-17

**Authors:** Damla Leman Bektasoglu, Semih Cakmak, Ahmet Kirgiz, Nilay Kandemir Besek, Burcin Kepezyildiz, Muhittin Taskapili

**Affiliations:** 1. Department of Ophthalmology, University of Health Sciences, Beyoglu Eye Training and Research Hospital, Istanbul, Turkey; 2. Department of Ophthalmology, Karapinar State Hospital, Konya, Turkey

**Keywords:** Anterior segment, COVID-19, lens, surgery, trauma

## Abstract

**Objectives:**

This study evaluated anterior segment surgeries performed during the coronavirus 2019 (COVID-19) pandemic. Prevention of virus transmission is a critical consideration for surgeons, and includes assessment of etiology, the referral region, demographic characteristics, and the surgery to be performed.

**Methods:**

The data of 144 patients who underwent anterior segment surgery between March 19, 2020 and June 1, 2020 were retrospectively reviewed. The patient demographic data and details of ophthalmological examination findings, the region patients were referred from, and the type of surgery performed were recorded and analyzed.

**Results:**

A total of 144 patients, 49 women (34%) and 95 men (66%), were included in this study. The mean age of the patients was 31.30±25.88 years (range: 1–86 years). The presenting complaint was in the right eye in 43.7% of the cases, in the left eye in 52.8%, and in both eyes in 3.5% of the cases. While 94.4% of the applications were from Istanbul, the remaining 5.6% were from outside the province. Though 43.7% of the cases were patients seen previously at the study hospital in Istanbul, 56.3% presented for the first time. This hospital was the first referral center in only 39.6% of the cases. Evaluation of etiology indicated that corneal perforation (18.1%) was the most common, followed by keratitis (13.2%). The most common surgical intervention applied was amnion membrane transplantation (19.4%), followed by perforation repair (16.7%).

**Conclusion:**

Ophthalmological surgeries continue to be performed during the ongoing COVID-19 pandemic, however, special algorithms must be used to reduce the risk of COVID-19 transmission and to ensure continuity of healthcare for ophthalmology patients.

## Introduction

Due to the outbreak of novel coronavirus (COVID, 2019-nCoV) that began in Wuhan, China, quarantine measures began worldwide and in Turkey. As a result of the government decision of March 19, 2020, elective surgeries were postponed throughout the country, especially in Istanbul, where the disease first started to appear, as in all over the world (1-3). In this context, eye surgeries were delayed to ensure that the intensive care units in hospitals work properly, to reduce bed occupancy rates, and to prevent congestion in hospitals. In addition, many ophthalmology clinics in Istanbul have been turned into COVID-19 clinics to provide an adequate number of beds for COVID-19 patients. Our clinic continued to work as a reference center during this period to perform emergency ophthalmologic surgeries in Istanbul.

During this extraordinary period when the whole world was caught unprepared, there were changes in our surgical approaches. Until then, there was no evidence-based guideline on an ophthalmic emergency case definition. The Turkish Ophthalmology Association (TOA) published a guideline on which ophthalmic surgeries should be considered as an urgent eye condition and which should be postponed (4). It is clear that it is not always easy to distinguish between emergency and elective cases in ophthalmology surgery (5, 6). Sometimes, the timing of surgery can affect the patient’s possible final visual acuity and risk of blindness (7, 8).

The aim of this study was to evaluate the anterior segment surgeries performed in our hospital in terms of etiology and surgical technique and to reveal the demographic characteristics of these cases during the period of restrictions due to the COVID-19 pandemic.

## Methods

This retrospective study was conducted by reviewing the archive files of the surgeries performed in the anterior segment unit of our hospital between March 19, 2020, and June 1, 2020. Ethical approval was obtained from the ethics committee of the Health Sciences University (2021/3, 3/7), and the study followed the tenets of the Declaration of Helsinki. Preoperatively, a brief overview of the surgery was given, and written informed consent was obtained from all the cases.

During the lockdown period, our hospital continued to perform surgeries. A number of measures have been implemented to reduce the risk of contamination for health-care professionals and patients while ensuring the continuity of the service. All cases admitted to our clinic were evaluated in terms of fever, symptoms of upper respiratory tract infection, and contact with suspected or confirmed COVID-19 cases before the surgery.

Surgical preparations were initiated by deciding the urgency of the operations to be carried out in line with the TOA guideline (4). The patients were evaluated, and surgeries were performed under local or general anesthesia. Surgeries performed on the cornea, lens, sclera, anterior chamber, and conjunctiva were included in the study. Patients who underwent vitreoretinal procedures and glaucoma surgeries were excluded from the study.

The age and gender of the patients, the region they were referred from, whether they had been followed up in our hospital before, the number of centers they applied to, and the operations performed were recorded. Visual acuities of the patients were evaluated using the Snellen chart at the pre-operative and post-operative final control examination. Detailed biomicroscopic and fundus examinations were noted, and intraocular pressures (IOPs) were measured.

Statistical analysis was performed using SPSS version 22.0 (SPSS Inc., Chicago, Illinois, USA), and descriptive statistical methods were used in the analysis of the data. Descriptive analyses were represented as number, percentage, mean, and standard deviation for continuous variables, and number, percentage, median, and standard error for categorical variables.

## Results

A total of 144 cases were included in the study, 49 women (34%) and 95 men (66%). The mean age of the patients was 31.30±25.88 (1–86) years. While the presenting complaint was in the right eye in 43.7% of the cases, it was in the left eye in 52.8% and in both eyes in 3.5% of the cases.

While 94.4% of all cases applied from Istanbul, the remaining 5.6% applied from outside the province. Whereas 43.7% of the cases consisted of the patients we had followed up previously, 56.3% presented to our hospital for the 1st time. Our hospital was the first referenced center in 39.6% of the cases, the second in 45.8%, the third in 11.1%, the fourth in 2.8%, and the fifth referenced center in 0.7%.

When evaluated in terms of etiology, corneal perforation (18.1%) was the most common followed by keratitis (13.2%) and intracorneal foreign body (11.8%). The diagnoses of the cases requiring surgery are shown in Table 1.

**Table 1.** The diagnoses of the cases requiring surgery on March-June 2020

**Diagnoses**	**n**	**%**
Corneal perforation	26	18.1
Keratitis	19	13.2
Intracorneal foreign body	17	11.8
Corneal suture after ocular surgery	14	9.6
Cataract	10	6.9
Corneal melting	10	6.9
Phacomorphic glaucoma	10	6.9
Persistent corneal epithelial defect	9	6.3
Scleral perforation	6	4.2
Conjunctival laceration	4	2.8
Loose sutures after PK	3	2.1
Wound dehiscence after PK	2	1.4
Granulation tissue of the conjunctiva	2	1.4
Suture exposure in scleral-fixated IOL implantation	2	1.4
Positive seidel test after ocular surgery, requiring corneal suturing	2	1.4
Anterior chamber dislocation of intraocular lens	2	1.4
Others (aphakia after lens removal, membranous conjunctivitis, iridodialysis, residual lens fragments on the anterior chamber, foreign body on the anterior chamber, giant papillae)	6	4.2

PK: Penetrating keratoplasty; IOL: Intraocular lens.

The most common (19.4%) surgical intervention was amnion membrane transplantation (AMT), followed by perforation repair (16.7%), and phacoemulsification and intraocular lens implantation (13.8%), respectively. Surgical interventions performed are shown in Table 2.

**Table 2.** Anterior segment surgeries performed from March to June 2020

**Surgical interventions**	**n**	**%**
Amniotic membrane transplantation	28	19.4
Perforation repair	24	16.7
Phacoemulsification with intraocular lens implantation	20	13.8
Removal of intracorneal foreign body	17	11.8
Removal of loose corneal suture	11	7.5
Corneal suturing	5	3.5
Intrastromal injection of the antibiotic agent	5	3.5
Examination under general anesthesia	5	3.5
Penetrating keratoplasty	5	3.5
Allogenic lenticule implantation for corneal perforation	4	2.8
Conjunctival suturing	4	2.8
Surgical repair of loose sutures with iwound separation after PK	4	2.8
Secondary IOL implantation	2	1.4
Excision of conjunctival granuloma	2	1.4
Management of suture exposure in scleral-fixated IOL implantation	2	1.4
Others (reposition of IOL, removal of anterior chamber foreign body, lens frag-ment removal from the anterior chamber, supratarsal steroid injection, conjunctival membrane removal)	6	4.2

PK: Penetrating keratoplasty; IOL: Intraocular lens.

## Discussion

The COVID-19 outbreak has changed not only the number of patients but also the nature of the surgeries performed in ophthalmology, as in many areas. In our hospital, arrangements were made for elective interventions in line with the TOA recommendations, and surgical interventions not included in the emergency category were postponed according to this guideline. However, as a result of the allocation of ophthalmology clinics to COVID-19 patients in many centers in Istanbul, an increase was observed in trauma patients referred to our clinic compared to previous periods.

In our center, several steps were taken to overcome the ongoing need for surgical intervention care during this epidemic period and to ensure that patients are examined and treated in a safe environment as possible. Each case was assessed for risk of visual loss and emergency. Operating room and emergency room teams working alternately were formed to prevent cross-contamination. Necessary applications were performed to provide environmental disinfection between operations performed on the same day.

The routes of transmission of COVID-19 are thought to include droplet, contact, and contaminated surfaces, as well as the ocular surface. Ophthalmologists are in the high-risk group in terms of transmission not only because they are in close proximity to patients during the examination, contact with conjunctiva and tears, but also because of the high daily number of outpatients and emergency patients (9, 10). Furthermore, studies suggest that virus particles can survive in droplets for a few hours and can survive on surfaces for several days (11). Therefore, to reduce human-to-human virus transmission in the struggle against the COVID-19 pandemic, national ophthalmology associations in many countries have recommended that any treatment other than emergency or urgent should be avoided. The frequencies and distributions of ophthalmological procedures performed during the pandemic have been discussed in the literature (1-3, 5, 6). However, as far as we know, there are no studies in the literature that evaluate epidemiologic and clinical features of a large patient series, as our patient series, during the lockdown period, and report anterior segment surgical interventions performed in Turkey.

In our study, when ophthalmic emergencies were examined in terms of etiology, corneal perforation was the most common by 18.1%. When the study conducted by Tang et al. (12) between February and April 2020 in a tertiary center in Hong Kong was evaluated in terms of anterior segment surgery, corneal perforation repair was the most common by 15.8%. Unlike the Tang et al. study, in which no evaluation was made in terms of etiology, in our study, corneal penetration was the most common etiology, but AMT was the most common procedure. The reason for this difference is that AMT was also applied in etiologies such as keratitis, spontaneous perforation, corneal melting, and persistent epithelial defect in our cases.

In a study in which all emergency ophthalmological surgeries were evaluated by Du et al. (6) in the period from December 2019 to March 2020, during which measures began to be taken after the first COVID-19 case, it was reported that the most common surgery was glaucoma surgery and eye traumas were rarely observed. The fact that our center is a tertiary branch hospital, the closure of ophthalmology departments due to COVID-19 in some of the other health institutions, and the high number of patients referred to our hospital within and outside the province are among the reasons for performing the high number of eye trauma surgeries during the quarantine period in our hospital.

Pediatric patients diagnosed with congenital cataracts, elderly and weak patients over the age of 70, and patients diagnosed with phacomorphic glaucoma constituted the group with phacoemulsification and intraocular lens implantation surgery performed in our clinic during this period. In ophthalmology practice, while the patient group undergoing elective surgery constituted the majority of the patient population undergoing cataract surgery, the reasons for not delaying surgery in these patients included the primary evaluation of pediatric patients due to the risk of amblyopia, uncontrollable IOP elevation due to phacomorphic glaucoma, and possible optic nerve destruction (7). In pediatric cataract cases, the timing of cataract surgery has significant effects on ultimate visual acuity, amblyopia management, and stereopsis (13, 14). In cases with phacomorphic glaucoma, phacoemulsification and IOL implantation are effective in improving visual acuity and in the control of IOP (15). Furthermore, in a study by Shih et al. (16) in which elective cataract surgery performed during the pandemic period was evaluated, it was reported that elective surgeries should continue even at low capacity in cases that may cause morbidity in phacomorphic glaucoma cases.

When the cases included in our study were examined in terms of the ranking of referral centers, it was observed that 61.4% of the cases were referred to our hospital after their application to two or more health institutions. In a study conducted by Al-Khersan et al. (17) in which emergency ocular surgeries performed between April 2019 and April 2020 were compared, it was reported that 36% of all patients who underwent ocular surgery were referred from an external center. Moreover, we considered that the reason 56.3% of the cases applied to our hospital for the 1st time was due to the closure of ophthalmology clinics of the surrounding centers.

This study has some limitations. First, this study has a retrospective design. Second, although the data were obtained from our center with the highest number of patients within the boundaries of Istanbul, they may not fully reflect the ophthalmology cases across the country. Furthermore, the results of this study may not completely apply to other countries in the world, where restrictions imposed during the quarantine period may differ.

The COVID-19 outbreak has significantly affected patient selection and surgical interventions in Turkey, our hospital, and all over the world. The number of surgical interventions performed in our hospital decreased significantly, and only emergency cases were intervened. Each patient was considered a potential carrier of COVID-19 and examined by taking necessary measures before and after surgery. While the first examination was performed in the isolation room in emergency cases known to have a positive COVID-19 test, maximum measures were taken to prevent contamination during and after surgery. While this period caused unique difficulties for patients and surgeons, we were able to provide safe surgical care for the patients in need with the arrangements we made in our hospital in accordance with the TOA recommendations.

## Conclusion

During this quarantine period, while corneal perforation was the most common indication among ocular emergencies requiring surgery, AMT was the most performed intervention. The struggle against COVID-19 is still ongoing. Special arrangements are required to reduce the risk of transmission for health-care professionals and patients and to ensure continuity of health-care services for ophthalmology patients. New emergency treatment algorithms can be developed for similar situations that may occur in the future.

### Disclosures

**Ethics Committee Approval:** Ethics committee of the Health Sciences University, Number of decisions 3/7, Registration number 21/10, Date: 22.01.2021.

**Peer-review:** Externally peer-reviewed.

**Conflict of Interest:** None declared.

**Authorship Contributions:** Involved in design and conduct of the study (AK, DLB, SÇ); preparation and review of the study (DLB, SÇ, AK, MT); data collection (DLB, SÇ); and statistical analysis (BKY, NKB, MT).

## References

[1] Sengupta S, Honavar SG, Sachdev MS, Sharma N, Kumar A, Ram J (2020). All India Ophthalmological Society–Indian Journal of Ophthalmology consensus statement on preferred practices during the COVID-19 pandemic.. Indian J Opthalmol.

[2] Safadi K, Kruger JM, Chowers I, Solomon A, Amer R, Aweidah H (2020). Ophthalmology practice during the COVID-19 pandemic.. BMJ open ophthalmology.

[3] Lai TH, Tang EW, Chau SK, Fung KS, Li KK (2020). Stepping up infection control measures in ophthalmology during the novel coronavirus outbreak: an experience from Hong Kong.. Graefes Arch Clin Exp Ophthalmol.

[4] Turkish Ophthalmological Association,Turkey (2020). Guideline of urgent ophthalmic surgeries 2020.. https://koronavirus.todnet.org/pandemi-nedeni-ile-acil-kabul-edilen-gz-ameliyatlar.

[5] Tognetto D, Pastore MR, De Giacinto C, Cecchini P, Agolini R, Giglio R (2020). Managing ophthalmic practices in a referral emergency COVID-19 hospital in north-east Italy.. Acta Ophthalmol.

[6] Du H, Zhang M, Zhang H, Sun X (2020). Practical experience on emergency ophthalmic surgery during the prevalence of COVID-19.. Graefes Arch Clin Exp Ophthalmol.

[7] Saxena R, Singh D, Jethani J, Sharma P, Sinha R, Sharma N (2020). Pediatric ophthalmology, strabismus and neuro-ophthalmology practice in the COVID-19 era: All India Ophthalmological Society guidelines.. Indian J Ophthalmol.

[8] Beğendi D, Duranoğlu Y (2018). Konjenital katarakt cerrahisinde temel prensipler ve göz içi lens seçimi.. Turkiye Klinikleri J Ophthalmol.

[9] Shah RD, Randleman JB, Grossniklaus HE (2012). Spontaneous corneal clearing after Descemet's stripping without endothelial replacement.. Ophthalmology.

[10] Bozkurt B, Eğrilmez S, Şengör T, Yıldırım Ö, İrkeç M (2020). The COVID-19 Pandemic: clinical ınformation for ophthalmologists.. Turk J Ophthalmol.

[11] Van Doremalen N, Bushmaker T, Morris DH, Holbrook MG, Gamble A, Williamson BN (2020). Aerosol and surface stability of SARS-CoV-2 as compared with SARS-CoV-1.. N Engl J Med.

[12] Tang EW, Wong DH, Chan YY, Li KK (2020). Emergency ophthalmic surgeries during COVID-19—a Hong Kong perspective.. Graefes Arch Clin Exp Ophthalmol.

[13] Kucuksumer Y, Utine CA, Perente İ, Kevser MA, Yilmaz ÖF (2006). Pediatric cataracts: our surgical approach.. Turk J Ophthalmol.

[14] Atalay HT, Gülpınar İkiz GD (2019). Factors Affecting the Visual Prognosis in Bilateral Infantile Cataracts.. Journal of Glaucoma-Cataract.

[15] Lee SJ, Lee CK, Kim W-S (2010). Long-term therapeutic efficacy of phacoemulsification with intraocular lens implantation in patients with phacomorphic glaucoma.. J Cataract Refract Surg.

[16] Shih KC, Wong JKW, Lai JSM, Chan JCH (2020). The case for continuing elective cataract surgery during the COVID-19 pandemic.. J Cataract Refract Surg.

[17] Al-Khersan H, Kalavar MA, Tanenbaum R, Lazzarini TA, Patel NA, Yannuzzi NA (2021). Emergent ophthalmic surgical care at a tertiary referral center during the COVID-19 pandemic.. American journal of ophthalmology.

